# Microscopical Memoranda

**Published:** 1874-03-15

**Authors:** 


### Microscopical Memoranda.

MIGRATION OF WHITE CORPUSCLES.—Dr. Thomas read, before the German Association of Naturalists at Wiesbaden, a paper on the migration of the white corpuscles into the lymphatics of the tongue of a frog. He injected the lymphatics of the living animal with an extremely dilute solution, not containing more than $\frac{1}{2000}$th to $\frac{1}{8000}$th part of nitrate of silver, and found that, with certain precautions, this did not lead to stasis of the blood in blood-vessels, but only to a lively exodus of the white corpuscles from their interior. After the lapse of some time, when the parts had begun to recover from the injurious effect of the injection, he was enabled to observe the re-entrance of the corpuscles into the lymphatic vessels, through certain stomata in their walls, now marked and rendered distinct by a precipitate of the silver salt. In a second series of researches the lymphatics were injected with a dilute emulsion of cinnabar, in a three-quarter per cent. solution of common salt. The cinnabar was in part deposited in the stomata of the lymphatics, and partly passed through them, and was deposited in the tissues in the form of small, round, cloudy patches. The evidence of the identity of the stomata, brought into view by means of the cinnabar, with those rendered evident by the nitrate of silver, is obtained by observing their peculiar grouping, and by the subsequent injection of nitrate of silver into the same vessels. The injection of the cinnabar causes very little disturbance of the circulation. If a lively exodus of the white corpuscles from the blood-vessels be produced by making an abrasion of the surface, the migrating cells quickly make their appearance in the stomata of the lymphatics marked out by the cinnabar. They then take up the particles of the cinnabar into their interior, which causes them to lose their activity and accumulate in the stomata. They then appear in the form of cauliflower excrescences, projecting into the interior of the lymphatics, which gradually break up into their constituent cinnabar-holding cells. These may be traced into the larger vessels, and from them into the blood. In these researches, a remarkable regularity, or uniformity, in the track pursued by the white corpuscles, was observed. They pass away from the blood-vessels nearly at right angles into the tissues, their course, however, being in a series of short zigzags. They all appear to travel about the same pace.—*Lancet.*—*Amer. Jour. Med. Sciences.*

THE TERMINATION OF NERVES IN SEBACEOUS GLANDS. — G. Colasanti (*Centralblatt*, No. 34, 1873) has followed out the distribution of the nerves in the Meibomian follicles of man, the ox, horse, and sheep, using for this purpose thin sections, stained with gold, as recommended by Conheim. As the result of his investigations, he says that, in successful gold-stained preparations, medullated nerve fibres may be detected in the connective tissue surrounding the gland. These fibres run in company with the blood capillaries, and are distributed with them. They give off smaller fibres, which run to the fundus of the follicles, perforate the membrana propria, and, losing their medullary sheath, break up into the primitive fasiculi, "presenting the well-known varicose aspect. These finer fibres form a plexus in the interior of the alveolus, and wind round the several gland epithelium cells. The plexus does not, however, extend into the excretory ducts. The nerves terminate in a precisely similar manner in the subaceous glands of the hair follicles."

It will be observed that these researches correspond very closely with Beale's views of the termination of nerve fibres, and directly contradict the statements of Pfluger, in *Stricker's Manual of Histology*, as to the termination of these nerves in the salivary glands.—*Lancet.*

---

DISTINGUISHING MAMMALIAN FROM REPTILIAN BLOOD. — R. M. Bertolet, M.D., Microscopist to the Philadelphia Hospital, refers to the great difficulty which is experienced in determining the kind of blood, by the ordinary methods of examination in medico-legal cases.

If examined with the microscope, as it is ordinarily found in the dried state, the corpuscles are shrivelled and deformed. The addition of water extracts the coloring matter, and though it causes them to swell up, does not restore them to their original condition. It causes the red corpuscles to lose their bi-concave shape and approach the spherical. The oval discs of reptiles, birds, etc., lose something of their peculiar shape, and become more like mammalian blood.

In moistening such blood he uses a solution of sulphate of soda, or, better still, slightly acidulated, pure glycerine. This preparation "is carefully irrigated with a properly prepared alcoholic solution of guaiacum resin; then, when a very small quantity of the ethereal solution of the peroxide of hydrogen (ozonic ether) is introduced beneath the glass cover," the red corpuscles are changed to an uniform color, which varies in the different corpuscles, "from a light sapphire to a deep indigo blue."

In the nucleated corpuscles of birds, reptiles, etc., however, "*the nucleus is seen as a sharply-defined, dark blue body, while the protoplasm surrounding it assumes a more delicate violet hue.*" The distinction between the two kinds of blood, by this means, is so plain as to be evident, even to an an ordinary gentleman of the jury.—*Amer. Jour. Med. Sciences*, Jan'y.

---

HÆMOPTYSIS FROM ANEURISM OF PULMONARY ARTERY.—Dr. Silver, in a meeting of the Pathological Society of London, showed a piece of lung, with an aneurismal dilatation of a small branch of the pulmonary artery, in a man who had had repeated attacks of hæmoptysis, and who died in one of them. A cavity was found at the apex, and a small aneurism.—*Lancet.*

---

BLOOD AND DEJECTIONS IN CHOLERA.—M. Hayden, of Paris, has been investigating this subject. He finds an increase in the number of the white corpuscles and fragments of the red corpuscles, which, he says, may be explained by the stasis during the algid stage, together with the decrease in the proportion of water. No fungi of any kind were found in the blood. He also found a certain amount of viscidity in the corpuscles, which he accounts for by the presence of carbonic acid gas. No other changes were observed. He examined the dejections for the cholera fungus, and found vibriones, as in other decaying animal matter; but the vibriones were not always present, and, when present, not always of the same kind. There were absent ten different kinds. He accordingly casts in his testimony with the already overwhelming load of evidence against the fungus origin of the disease.

The *post-mortem* changes that were constant were confined to the intestinal canal, and could not be distinguished, except in severity, from ordinary intestinal catarrh.—*Lancet.*

NEW RESEARCHES ON INFLAMMATION.—Prof. Conheim, in a recently published article, states that inflammation consists in some local change of the vessels of the affected part, and not in their dilitation with the accompanying increase in the rapidity in the flow of the blood.—*Brit. Med. Jour.*

